# International cohort study for pediatric Behçet’s Disease: update 2011

**DOI:** 10.1186/1546-0096-9-S1-O32

**Published:** 2011-09-14

**Authors:** I Koné-Paut, M Darce Bello, F Shahram, M Gattorno, R Cimaz, B Chkirate, K Bouayed, M Hofer, S Ozen, J Anton, I Tugal Tutkum, J Kümmerle-Deschner, S Benamour, S Assad Khalil, L Cantarini, A Faye, A Letierce, TA Tran, F Davatchi, O Kasapcopur, A Al Mayouf, C Pajot, S Nielsen, L Lepore, W Bono

**Affiliations:** 1 Bicêtre University Hospital, France; 2 Shariati Hospital, Tehran, Iran; 3 Gaslini Institut. Genoa, Italy; 4 Meyer Institut Florence, Italy; 5 Rabat University Hospital, Morocco; 6 Vaudois University Hospital Lausanne. Switzerland; 7 Hacettepe University Ankara, Turkey; 8 E Llobregat Hospital Spain; 9 Istanbul University, Turkey; 10 University of Tuebingen, Germany; 11 IBN Rochid University Hospital , Casablanca, Morocco; 12 Alexandria Faculty of Medicine, Alexandria, Egypt; 13 University of Siena, Siena, Italy; 14 Robert Debré University Hospital, France; 15 URC, Bicêtre University Hospital, France

## Background

Behçet Disease (BD) is exceptionally observed in children and raises diagnosis problems because there is no specific biologic marker. Sets of clinical criteria have been proposed for adult patients only.

## Aim

Is to set-up an international cohort of patients suspected with BD and selected on homogenous criteria. The cohort is aimed at defining an algorithm for definition of the disease in children, reflecting the natural history.

## Methods

Centers specializing in PedBD have been called to collaborate documenting their patients into a single online database. An international expert committee has defined inclusion criteria as follows: first sign before 16 years, new patient or patient followed since less than 3 years, patient followed 4 years, consent obtained, and recurrent oral aphthosis [OA] (more than 3 attacks/year) associated to at least one of following symptoms: genital ulceration, erythema, folliculitis, pustulous/acneiform lesions, positive pathergy, uveitis, venous/arterial thrombosis, family history. Data are updated every year and patient’s files are classified by the expert committee into 3 groups: definite, probable and not BD. Statistical analyses are performed to compare the 3 groups.

## Results

In April 2011, 171 patients (85M/86F) from 20 centres of 11 countries have been included. Mean age at inclusion was 12.4y, at first symptom 7.4y and at BD suspicion 11.3y. 44 % of patients had only 1 symptom associated with OA, 27% had 2 and 29% had at least 3. 166 first visits have been done, 85% were receiving treatment; 26% had a family history of BD, 22% were HLAB51. 102 patients underwent first year visit, 65 had no new symptom, 25 had one, 11 had 2 and 1 had 3. 51 two year visits have been done, 40 patients had no new symptom, 12 had 1 and 6 had 2. The expert committee has examined 119 files (in which 13 twice) and classified 63 as definite and 54 as probable. Two were classified as not BD. 50/63 definite BD fulfilled the ISG criteria and 13/54 probable BD meet the international criteria.

## Conclusion

There is an increase of the number of patients classified in the BD group although they do not fill the international BD classification criteria.

